# Population Connectivity and Traces of Mitochondrial Introgression in New Zealand Black-Billed Gulls (*Larus bulleri*)

**DOI:** 10.3390/genes9110544

**Published:** 2018-11-09

**Authors:** Claudia Mischler, Andrew Veale, Tracey van Stijn, Rudiger Brauning, John C. McEwan, Richard Maloney, Bruce C. Robertson

**Affiliations:** 1 Department of Zoology, University of Otago, Great King Street, Dunedin 9016, New Zealand; bruce.robertson@otago.ac.nz; 2 Department of Environmental and Animal Sciences, Unitec, 139 Carrington Road, Auckland 1025, New Zealand; andrew.j.veale@gmail.com; 3 AgResearch Limited, Invermay Agricultural Centre, Private Bag 50034, Mosgiel 9053, New Zealand; tracey.vanstijn@agresearch.co.nz (T.v.S.); rudiger.brauning@agresearch.co.nz (R.B.); john.mcewan@agresearch.co.nz (J.C.M.); 4 Department of Conservation, 161 Cashel Street, Christchurch 8011, New Zealand; rmaloney@doc.govt.nz

**Keywords:** genetic diversity, population structure, endemic, mitochondrial introgression, isolation-by-distance

## Abstract

Black-billed gulls (*Larus bulleri*) are endemic to New Zealand and are suspected to be undergoing substantial population declines. They primarily breed on open gravel beds in braided rivers of the South Island—a habitat that is diminishing and becoming increasingly modified. Although management of this species is increasing, little has been published on their movements and demographics. In this study, both mitochondrial DNA (mtDNA) control region domain I and nuclear single nucleotide polymorphisms (SNPs) were examined to help understand the connectivity and population structure of black-billed gulls across the country and to help inform management decisions. Mitochondrial DNA showed no population structure, with high haplotype and low nucleotide diversity, and analyses highlighted mitochondrial introgression with the closely related red-billed gulls (*Larus novaehollandiae scopulinus*). Nuclear DNA analyses, however, identified two groups, with Rotorua birds in the North Island being distinct from the rest of New Zealand, and isolation-by-distance evident across the South Island populations. Gene flow primarily occurs between nearby colonies with a stepwise movement across the landscape. The importance from a genetic perspective of the more isolated North Island birds (1.6% of total population) needs to be further evaluated. From our results, we infer that the South Island black-billed gull management should focus on maintaining several populations within each region rather than focusing on single specific colonies or river catchments. Future study is needed to investigate the genetic structure of populations at the northern limit of the species’ range, and identify the mechanisms behind, and extent of, the hybridisation between red-billed and black-billed gulls.

## 1. Introduction

The dynamics of gene flow, dispersal, and the potential causes of population structuring are important to understand when creating a management plan for a species [1]. Population genetics aids in determining the presence or absence of distinct gene pools [2], providing insights into genetic variability within and between populations, and highlighting the ability of these groups to respond to declines over the long term [3,4,5].

Black-billed gulls/tarāpuka (*Larus bulleri*) are endemic to New Zealand and exist primarily in the South Island [6], with only approximately 1.6% of the population inhabiting the North Island [7]. North Island colonies appear to be a recent expansion, with the Rotorua breeding colony only establishing since the 1930s. Since 2012, the New Zealand Threat Classification System has listed the species as ‘Threatened, Nationally Critical’, due to suspected rapid population declines [8]. The gulls are unusual in that they mainly use unvegetated areas on braided rivers as breeding sites, but they also nest at river mouths, estuaries, lakes, and harbours [6]. Colonies are very densely populated, with potentially either tens or thousands of pairs in a single colony [6,9]. Unlike the closely related red-billed gull/tarāpunga (*Larus novaehollandiae scopulinus*), which primarily forages around marine environments, black-billed gulls mostly feed on terrestrial invertebrates, such as earthworms, larvae, and insects from ploughed farmlands, wet grass fields, and on mown hay meadows [6,10]. Patchy and unpredictable food supplies, as well as unstable nesting habitats, have resulted in adaptations, such as flock feeding near colonies, desertion of nesting sites if a food source runs out, and stronger group adherence rather than site faithfulness to maintain safety in numbers [11,12]. In the non-breeding season, birds congregate along the coast or remain in flocks inland [6]. Birds along the southern end of the North Island are assumed to be from the South Island, and birds in the Firth of Thames of the North Island are assumed to be from Rotorua and Gisborne [13].

Overall, few studies have been conducted on black-billed gulls, and the movements of individuals throughout the year are poorly understood [14]. Limited available banding information shows some individuals move between river catchments, as well as undertaking long-distance dispersal between the North and South Islands [15,16,17,18], but dispersal patterns and population connectivity remain unclear.

Demographic changes affect mitochondrial DNA (mtDNA) and nuclear DNA markers differently, due to their manner of inheritance and recombination [19,20]. Due to the complexity and high variability associated with genetic markers when examining population-level differences, mtDNA and nuclear DNA do not always show the same population structure, hence using both markers provides broader insight than using only one [2]. Hybridisation with red-billed gulls has previously been observed [13], and using multiple markers can reveal aspects, such as historical mitochondrial introgression, that may otherwise not be noticed [21]. Introgression is the exchange of genetic material between two species through hybridisation [22], and is particularly common in *Larus* gulls [23,24].

Genetic studies are increasingly being used to elucidate the population connectivity and ecology of seabirds, and the results from these studies are being applied in conservation management [25,26,27,28,29]. These studies of seabird genetics can reveal contrasting patterns of nuclear and mitochondrial divergence, with high genetic population structure indicating significant differentiation nearing species level segregation despite minimal morphological divergence (e.g., [30]). Since genetic studies of black-billed gulls have never been conducted, movements of birds are not well understood, and management of the species is increasing, here we assessed the patterns of population structure in black-billed gulls. Using both mtDNA and nuclear DNA, we examined the genetic structure of this endemic species using samples from across its range in New Zealand. We examined the potential for gene flow among gull populations, which would be consistent with the observation of dispersal between river catchments and between the North and South islands of New Zealand [15,16,17,18].

## 2. Materials and Methods

### 2.1. Sample Collection

Blood samples from black-billed gull chicks were obtained in December and January during 2014/2015, 2015/2016, and 2016/2017. Sampling was carried out under collaboration with the Department of Conservation (Christchurch, New Zealand) following their approved best practice guidelines. Chicks were targeted as it is too disruptive to a breeding colony to capture adults, due to the high density of nests. Only one chick was sampled per nest to avoid sampling siblings. Blood (<20 µL) was taken from the brachial vein in the wing with a sterile needle (26G × 1/2″), collected in a glass capillary tube, and stored in 0.5 mL of Queen’s lysis buffer solution (10 mᴍ Tris, 10 mᴍ NaCl, 10 mᴍ Na-EDTA, 1% n-lauroylsarcosine; pH 7.5) [31].

### 2.2. DNA Extraction and Sequencing

#### 2.2.1. Mitochondrial Control Region

**Figure 1:**
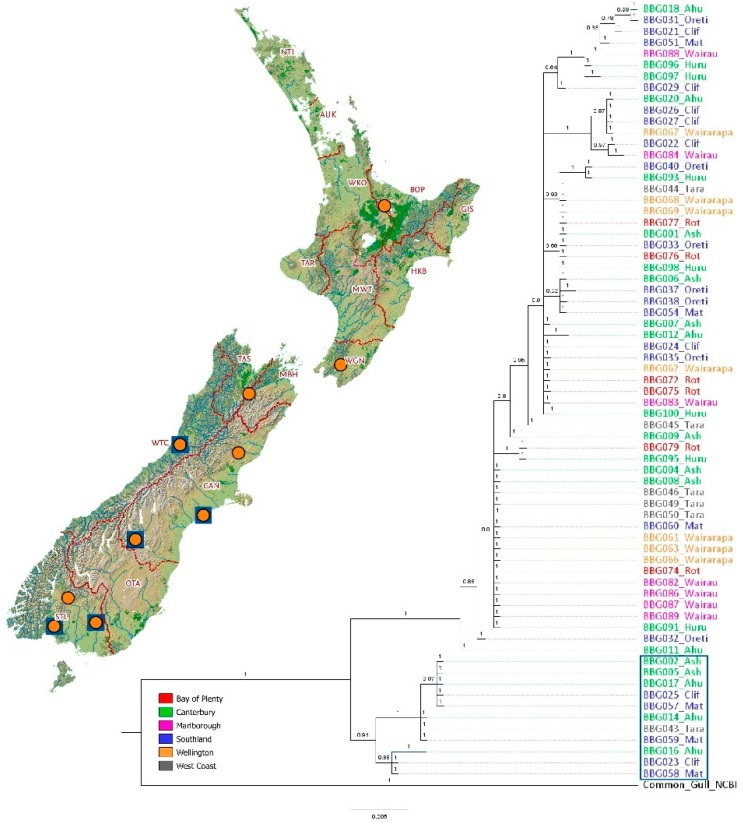
Bayesian tree of black-billed gulls using mtDNA (mitochondrial DNA) control region, first domain. Posterior probabilities are shown above the branches. Scale depicts the distance corresponding to 0.005 nucleotide substitutions per site. Orange circles show locations of colonies from which chick blood samples were analysed (10 colonies, 100 samples; 94 for Single Nucleotide Polymorphisms (SNPs), 69 for mtDNA control region). Blue rectangle on the tree contains locations corresponding to the blue squares on the inset map highlighting the locations of colonies found within the smaller clade (matching published red-billed gull data). Colour legend indicated regions. Regions are outlined: AUCK—Auckland, BOP—Bay of Plenty, CAN—Canterbury, GIS—Gisborne, HKB—Hawke’s Bay, MGH—Marlborough, MWT—Manawatu—Wanganui, NTL—Northland, OTA—Otago, STL—Southland, TAR—Taranaki, TAS—Tasman, WGN—Wellington, WKO—Waikato, WTC—West Coast.

A phenol-chloroform extraction [32] was used to extract genomic DNA from 100 blood samples chosen from 10 colonies across the range (Figure 1). Detailed methods for primer design and sequencing are given in Supplementary Material (Text S1). Briefly, the control region was amplified using primers BBG_CR_ND6_1F (5′-CCCCAGAACAAAACACAACC-3′) and BBG_CR_12S_1R (5′-CCCGCTCCTCTCTCCTTAGT-3′). Purified PCR products using Acroprep 96 filter plates (PALL Corporation, Port Washington, NY, USA) were sequenced with BigDye Terminator v.3.1 (ThermoFisher, Waltham, MA, USA) on an ABI 3730xl DNA sequencer (Applied Biosystems Inc., Foster City, CA, USA). Two internal control region primers, BBG_CR_INT_R1 (5′-GCCCTGACATAGGAACCAGA-3′) and BBG_GOOSEHAIRPIN_F1 (5′-ACATCCCTCCCCAACACAT-3′), were used to sequence a 597 base pair (bp) fragment of the first domain of the control region.

Sequences were aligned with Geneious^®^ 9.0.5 [33]. Of the 100 extracted DNA samples, 69 samples amplified successfully. The 221 bp D-loop domain region I sequence of the red-billed gull (NCBI Accession No.: AY584133.1) and a 430 bp *L. bulleri* control region sequence (NCBI Accession No.: FM209657 used as a comparison to our sequences) was aligned with the black-billed gull sequences in Geneious^®^. The red-billed gull sequence was used to determine how closely this species matched the black-billed gull.

#### 2.2.2. Mitochondrial Cytochrome b

The mtDNA control region analyses showed the presence of two major clades, a small clade separated from a large clade without any geographical pattern (Figure 1). To understand this result and determine if it is, due to the presence of nuclear copies of mitochondrial genomes or indicative of introgression, the cytochrome b gene was examined in a selection of seven samples; four from the small clade and three from the large clade (detailed methods are outlined in Supplementary Material, Text S2). A ~1000 bp fragment containing the entire cytochrome b gene was amplified using PCR primers H16064 (5’-CTTCAGTTTTTGGTTTACAAGACC-3’) and L14764 (5’-TGRTACAAAAAAATAGGMCCMGAAGG-3’) [33]. Purified PCR products (Acroprep 96 filter plates, PALL Corporation, Port Washington, NY, USA) were sequenced (BigDye Terminator v.3.1, ThermoFisher, Waltham, MA, USA) on an ABI 3730xl DNA analyser (Applied Biosystems Inc., Foster City, CA, USA) with one primer, L14764.

Sequences were aligned in Geneious^®^ 9.0.5 [33]. Sequences for all seven samples were aligned with a 1143 bp cytochrome b sequence of the red-billed gull (NCBI Accession No.: FM209918) and a 1143 bp cytochrome b sequence of a black-billed gull (NCBI Accession No.: FM209900.1 used as a comparison to our sequences) to determine the presence of introgression. This alignment was used to produce a neighbor-joining tree in Geneious^®^.

#### 2.2.3. Single Nucleotide Polymorphisms

Single nucleotide polymorphisms (SNPs) may include variation at many thousands of loci, thereby giving a detailed estimate of genome-wide variation. We used a single enzyme digest Genotyping by Sequencing (GBS) approach to generate SNP data at AgResearch’s Animal Genomics Group laboratory (Mosgiel, New Zealand). A total of 94 (plus two controls for data quality assurance) of the 100 phenol-chloroform extracted DNA samples mentioned above for the mtDNA control region were screened.

Bioanalyser traces (2100 Bioanalyser, Agilent Technologies, Santa Clara, CA, USA) indicative of single sequences in the region of interest were used to determine the best restriction enzyme to use for digesting genomic DNA. For black-billed gulls, the restriction enzyme ApeKI (R0643L, New England Biolabs, Ipswich, MA, USA) was selected, and sample-specific barcode adapters were ligated to the sticky ends of fragments after digestion. Individually barcoded samples were pooled to create a single library, which was followed by multiple PCRs. A column purified pooled library of the PCRs was then size selected (193–313 bp size range) via a Pippin (SAGE Science, Beverly, MA, USA; 2% agarose, dye-free with internal standards CDF2050, Marker L CDF2010). Sequencing of library was done on Illumina HiSeq2500 with single-end reads, and 101 cycles in high-output mode (v4 chemistry) [34].

A second GBS library was constructed due to a low sequencing depth using the above method. The size range used on the Pippin window was modified to 193 to 255 bp to give a subset of DNA fragments and therefore an increase in sequencing depth. This was run as an additional lane on the Illumina HiSeq2500 under the same conditions outlined above.

Methods outlined by reference [34] were followed for quality checks and adapter removals. In summary, FastQC v.0.10.1 [35] was used to check raw FastQC files. After adapters were removed with cutadapt [36], a random 10,000 reads per lane were checked for contamination using BLAST (https://blast.ncbi.nlm.nih.gov) against the NR database (ftp://ftp.ncbi.nlm.nih.gov/blast/db). A SNP loci catalogue was created as outlined in references [34,37]. UNEAK Tassel v.3.0.170 [38] trimmed barcodes, sequences with a second cut site, and common adapters from the raw reads; trimmed tags to 64 bp; grouped matching tags into tag pairs; and arranged mismatched tag pairs as candidate SNPs. UNEAK settings were: (i) -UFastqToTagCountPlugin -c 1 -e ApeKI selected reads with a barcode, cut site, and No missing data; (ii) -UMergeTaxaTagCountPlugin -m 600000000 -x 100000000 -c 5 ensured that a tag must be present at least five times to be output thereby removing rarities from sequencing errors; (iii) -UTagCountToTagPairPlugin -e 0.03 allowed an error rate of up to 0.03 when identifying SNPs with 1 bp mismatch; and (iv) -UMapInfoToHapMapPlugin -mnMAF 0.03 -mxMAF 0.5 -mnC 0.1 specified HapMap outputs to fall within an allele frequency of 0.03 to 0.5, with a minimum call rate of 0.1.

Kinship using GBS with Depth adjustment (KGD; https://github.com/AgResearch/KGD) was used for additional quality control, following outlines by reference [39]. Allele frequencies and sequence depths were evaluated, and SNP call rates and their minor allele frequencies (MAFs) were calculated. A fin-plot of MAFs versus Hardy-Weinberg disequilibrium (observed frequency of the reference allele homozygote minus its expected value) highlighted SNP average depth and pinpointed SNPs with non-Mendelian inheritance. High-depth and high MAF are assumed to represent genome duplication or repetitive regions [34]. Consequently, SNPs with Hardy-Weinberg disequilibrium below −0.05 were removed to prevent including SNPs that were not assembled properly.

SNPs were filtered at three different levels where 5%, 10%, and 25% of missing data were permitted per locus. Individuals were also filtered, so that only individuals with >50% of the SNPs were retained. Genotypic data were converted for subsequent population genomic analyses using PGDSpider v. 2 [40].

### 2.3. Data Analysis

#### 2.3.1. Mitochondrial Control Region

For phylogenetic analyses, the Akaike information criterion (AIC) in JModeltest2 [41] was used to determine the best fit model for sequence evolution (TVM + G). The population structure was examined with maximum-likelihood in IQ-TREE 1.6.3 [42,43,44] with 1000 bootstrap replicates to determine node support, and MrBayes v.3.2.1 was used to construct a Bayesian tree [45]. The Monte Carlo Markov Chain (MCMC) analysis of phylogeny in the MrBayes software carried out two independent runs for two million generations, sampling every 500 generations following a burn-in of 25%. All trees were visualised and adjusted in FigTree v.1.4 [46], and rooted with the *Larus canus* (NCBI Accession No.: AB208768) outgroup. A median-joining network to explore relationships amongst haplotypes was created using PopART 1.7 (popart.otago.ac.nz).

Statistical analyses were carried out in DnaSp v.6.1 [47] between river catchments, North versus South Islands, and overall. Nucleotide diversity (*π*) and haplotype diversity (*ɧ*) provide insight into genetic diversity. All 69 sequenced individuals were initially included in the calculations; however, once introgression was identified, the calculations were rerun using only the individuals in the large clade (58 individuals; Figure 1) to prevent skewing of values caused by hybridisation with red-billed gulls.

#### 2.3.2. Single Nucleotide Polymorphism Genomics

We used the R package *heirfstat* v.0.04-22 [48] to calculate observed and expected heterozygosity, and the allelic richness per locus, per sampling site, and across all samples. fastSTRUCTURE v.1.0 [49] was used to determine genetic structure among the different catchments. *K* was set from 1 to 8, and default parameters were used along with a logistic prior. The chooseK.py command selected the most suitable number of model components to explain any genetic structure [48]. DISTRUCT v.1.1 was used to visualise the clusters [50].

The R package *adegenet* [51] was used to run discriminant analysis of principal components (DAPC) retaining 30 principle components. Data were pooled per catchments before running the program to construct and visualise the composition of DAPC clusters. We used DAPC both to visualise the relationships between sampling locations, and in a separate analysis to determine the optimal number of clusters. Using the sampling locations as a priori clusters, we looked at the rates of individual assignment to each sampling location.

The R package *diveRsity* v.1.9.9 [52] was used to calculate the pairwise population and locus-specific F_ST_ [53]. Arlequin v.3.5 [54] tested the hypothesis of departure from panmixia and hence the statistical significance of each pairwise F_ST_ value between catchments with 110 random permutations. The pairwise population F_ST_ values were then run and visualised in SplitsTree4 [55] to create a two-dimensional UPGMA (unweighted pair group method with arithmetic mean) tree [56] based on 51,811 SNPs. The Isolde program in Genepop v.4.2 tested for isolation-by-distance (IBD), both for the complete dataset and on a reduced dataset consisting of only the South Island sampling locations [57,58].

## 3. Results

### 3.1. Mitochondrial Control Region

#### 3.1.1. Sequencing and Phylogenetic Analysis

**Figure 2:**
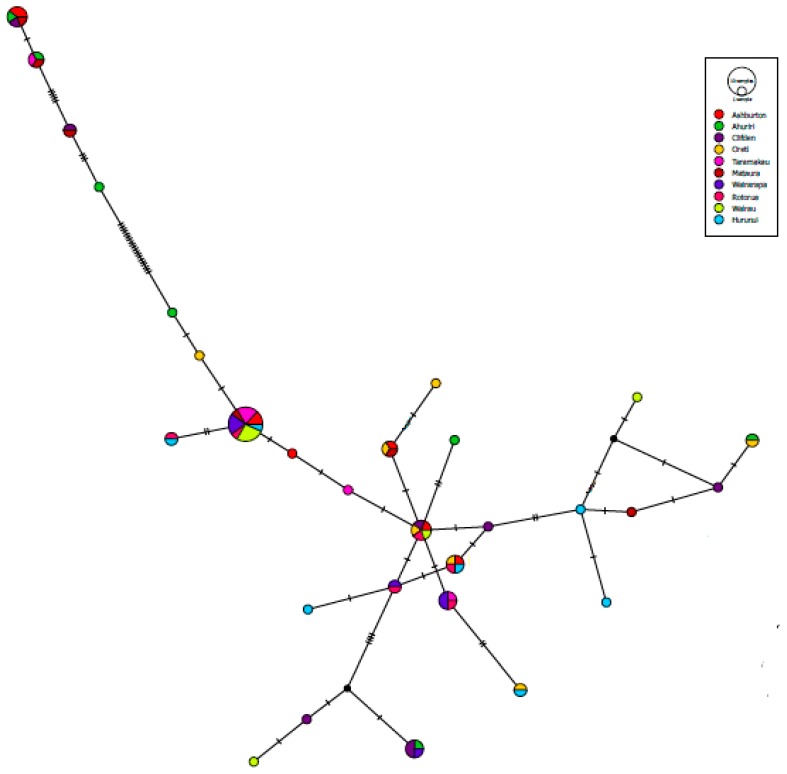
Median-joining haplotype network of black-billed gulls using 597 bp of mtDNA control region (first domain) sequences. Shared haplotypes are indicated by circles which are proportional to the number of individuals sharing that haplotype. Cross-hatches are indicative of the number of nucleotide differences between haplotypes. Colours show individual catchments.

There were 69 individuals for which mtDNA control region domain I sequences were obtained. The fragments consisted of 597 bp with 29 haplotypes. Bayesian and likelihood analyses of the mtDNA sequences showed no distinct genetic split between North versus South Island, between regions (Bay of Plenty, Wellington, Marlborough, West Coast, Canterbury, and Southland), or between river catchments (Rotorua, Wairarapa, Wairau, Taramakau, Hurunui, Ashburton, Ahuriri, Mataura, Oreti, and Waiau). Trees for IQ-TREE and MrBayes were similar; therefore, only the Bayesian tree is shown along with posterior probability (Figure 1). The analyses revealed two major clades but without a specific geographic pattern, with the larger of these two clades containing 58 individuals and the smaller one containing 11 (Figure 1). The individuals in the large clade closely matched the published *L. bulleri* control region sequence (NCBI Accession No.: FM209657; sequence lengths trimmed to 430 bp), with the most commonly observed haplotype (BBG004_Ash) in this clade differing from the published sequence by one bp. The individuals in the smaller of these two clades closely matched the published red-billed control region sequence (NCBI Accession No.: AY584133.1; sequence lengths trimmed to 221 bp) with the most commonly observed haplotype (BBG002_Ash) in this clade also differing from the published red-billed gull sequence by one bp. The median-joining haplotype network showed the same split with 17 mutational steps between the two main clades with no clear separation of specific river catchments (Figure 2). The most frequently shared haplotype overall (shown as BBG004_Ash; Figure 2) was found in seven catchments/six regions and therefore exists across the entire New Zealand geographic range (i.e., both North and South Islands).

Once introgression was determined, the 11 individuals from the smaller of the two major clades (Figure 1) were removed from genetic variation (haplotype and nucleotide diversity) calculations to prevent skewing of values caused by hybridisation with red-billed gulls. When examining number of haplotypes within regions for the large clade birds only, Canterbury has 14 haplotypes, and Southland region has 13, with the North Island having seven and the South Island having 24 (Table 1). Regionally, haplotype diversity ranges from 0.700 in West Coast to 1.000 in Bay of Plenty. The North Island has a lower overall haplotype diversity (0.872) than the South Island (0.927). Nucleotide diversity is between 0.003 in West Coast to 0.008 in Canterbury and Southland, and the North Island has a slightly lower nucleotide diversity (0.006) than the South Island (0.007).

#### 3.1.2. Mitochondrial Cytochrome b

No detailed analyses were done on the mitochondrial cytochrome b sequences. The seven cytochrome b sequences were examined to assist the explanation behind the two main clades seen in the Bayesian tree (Figure 1), specifically to determine if the smaller of the two main clades was due to the presence of nuclear copies of mitochondrial genomes or indicative of introgression. The neighbour-joining tree of cytochrome b (Figure S1) showed the same split as was seen for the corresponding control region sequences. Sequences of birds from the small clade (Figure 1) aligned with published red-billed gull, and sequences of large clade birds aligned with published black-billed gull (detailed in Supplementary Material, Text S3).

#### 3.1.3. Single Nucleotide Polymorphisms

Unfiltered, 402,672 SNPs were obtained for 94 individuals, and contamination was not detected using the BLAST contamination check. Three different levels of filtering were performed where 5%, 10%, and 25% missing data were permitted per locus, giving a total of 15,095, 51,811, and 176,298 SNPs retained, respectively. The performance and outputs of all three datasets were compared with multiple analyses and the results were essentially identical: Pairwise F_ST_ values between populations were identical when regressed (y = 0.9327x − 5 × 10^−6^ R^2^ = 0.9983); the same number of optimal clusters were recorded in both fastSTRUCTURE and in DAPC; and clustering plots were visually indistinguishable. Therefore, values obtained with the 10% missing data per locus are presented throughout for consistency. The 10% missing data were chosen to represent the middle value of the three percentages (5%, 10%, 25%).

**Figure 3:**
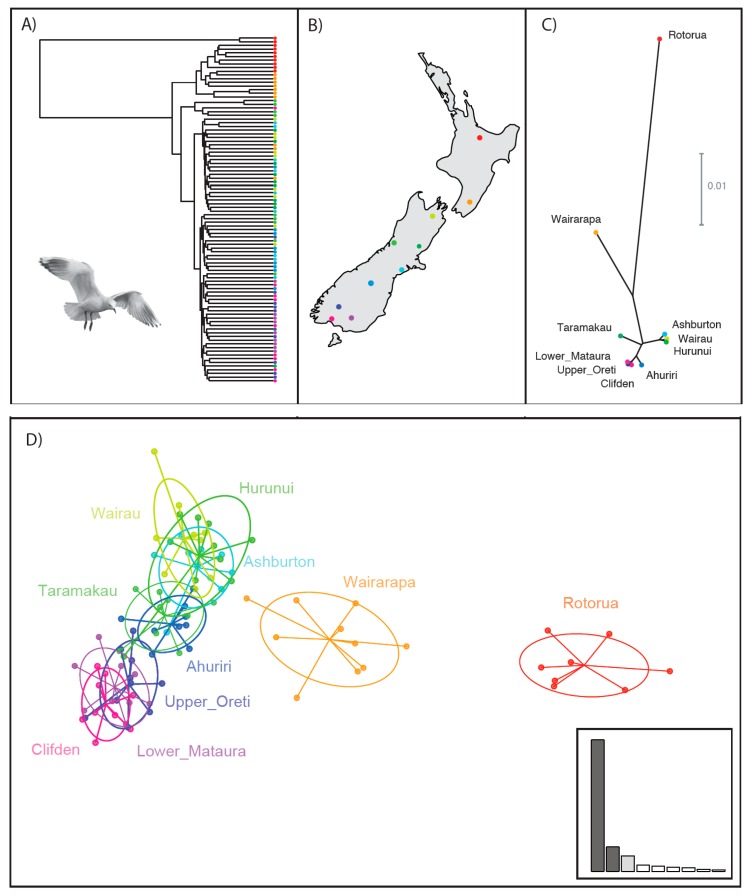
(**A**) Neighbour-joining tree of black-billed gulls from across New Zealand using SNPs based on relatedness estimates derived from Kinship using GBS with Depth adjustment (KGD). Colours correspond to location of sampled catchments in map inset (**B**). (**C**) Unweighted Pair Group Method with Arithmetic Mean (UPGMA) tree showing population divergence of black-billed gulls in New Zealand as measured by F_ST_ from SNPs. (**D**) Discriminant analysis of principal components (DAPC) scatterplot of SNPs from 10 black-billed gull colonies sampled across New Zealand. Individuals are shown as dots with the majority grouped within ellipses. Inset shows the proportion of variation accounted for by each PC.

Based on the relatedness estimates derived from KGD, two individuals from Rotorua were highly divergent from all others sampled and appeared to be divergent enough to be red-billed gulls that had mistakenly been sampled (Figure 3A). This possibility was discussed with the collector of the Rotorua blood samples, who confirmed that it was possible that these individuals were misidentified, since Rotorua is a mixed colony of red-billed and black-billed gulls. A mtDNA control region sequence was obtained (and excluded from all mtDNA analyses) for only one of these two individuals (BBG078_Rot), and this sequence matched with the red-billed gull. Given the extreme divergence of these two individuals and the potential biases caused by including misidentified individuals from a different species, they were excluded from all subsequent analyses on genetic diversity, clustering and population connectivity. Unlike these two individuals, the 11 birds in the small clade of Figure 1 that were indicative of introgression when mitochondrial markers were analysed did not diverge in the relatedness estimates derived from KGD. This suggests mitochondrial capture, where the presence of introgression is visible only in mitochondrial markers but is not observed in nuclear markers.

The average observed, and expected, heterozygosity was similar across sampling locations, though the Rotorua sampling site had lower allelic richness than the other populations, with a general trend of lower diversity present in the North and higher in the South Islands (Table 2).

The neighbour-joining tree of individuals created from SNP data splits the North and South Islands river catchments into two major clades (Figure 3A). Average pairwise relatedness was significantly higher in the North Island group—particularly within the Rotorua samples. Within the South Island clade, there are several additional clades, but branch lengths between clades are short. A probable IBD pattern is visible in Figure 3, with lower latitude populations (red/orange) at the top, moving down to higher latitude populations (blue/purple) at the bottom (Figure 3A).

Genetic variation between river catchments as measured by F_ST_ was generally low, ranging from 0.002 and 0.043 (Table 3). The highest values were between Rotorua and Clifden (0.043)—which are also the most geographically distant. The two North Island river catchments of Rotorua and Wairarapa had higher pairwise F_ST_ values (0.011 to 0.043) than the South Island river catchments (0.002 to 0.011). All North Island values were significantly different from the South Island. The lowest F_ST_ values with a *p*-value <0.05 were between Hurunui and Ahuriri (0.004) and between Taramakau and Hurunui (0.004).

The UPGMA tree created by F_ST_ measurements shows three clear groupings (Figure 3C). Rotorua is highly divergent from the rest of the catchments, while Wairarapa is also separated but not as strongly as Rotorua. Taramakau and Ahuriri are removed from other South Island catchments. Wairau, Ashburton, and Hurunui are catchments on the east coast of the South Island, and group together. Clifden, Oreti, Mataura, which are southern South Island catchments, also group together. Highly significant levels of IBD were found across all catchments (North and South Islands) (*p* < 0.0001), and when tests were conducted among the South Island populations (excluding Rotorua and Wairarapa), the IBD values remained significant (*p* = 0.002; Figure S2).

**Figure 4:**
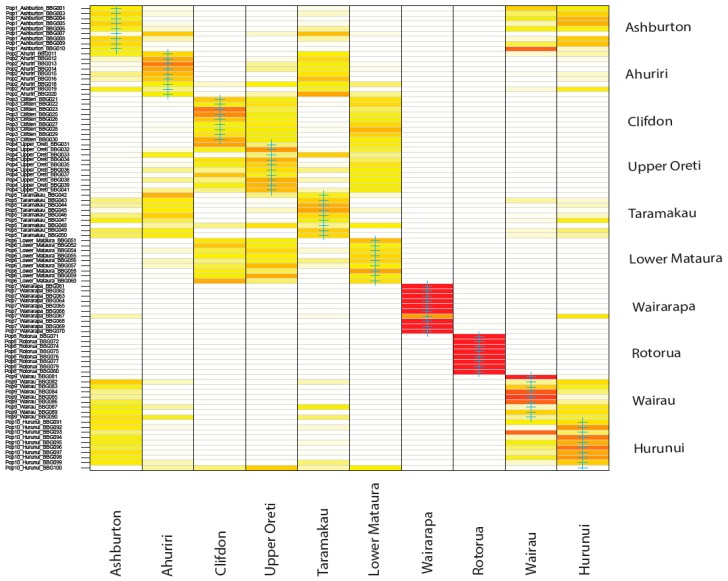
Discriminant analysis of principal components assignment plot showing the cluster membership probability for each of the black-billed gulls to each of the 10 sampled populations. Red indicates high assignment probability, white indicates low assignment probability, and yellow across multiple populations indicates assignment is mixed or uncertain.

The optimal number of clusters identified using DAPC analyses supported two clusters (Figure 3D). Rotorua (North Island) was identified as one cluster, and the eight South Island catchments along with Wairarapa (North Island), as another cluster. There was some support for a third cluster, which would isolate the Wairarapa catchment. Looking at the DAPC visualisation when each sampling location is treated as an a priori cluster, PC1 clearly differentiates the North Island sites from the South Island sites, while PC2 appears to show the isolation by distance pattern across the South Island. There appears to be slight grouping among the South Island, with Clifden, Oreti, and Mataura overlapping in the southern part of the island, Taramakau and Ahuriri overlapping in the centre, and Ashburton, Hurunui, and Wairau overlapping in the north. Using DAPC to assign individuals to these predefined sampling site clusters, showed that individuals from both Rotorua and Wairarapa all strongly assigned to their sampling site origin. For all other sites, assignment was less certain, with assignment appearing to overlap between Hurunui, Wairau and Ashburton in one group, Ahuriri and Taramakau in a second group, and Upper Oreti, Lower Mataura and Clifden forming a third group (Figure 4). These results are consistent with mixing between geographically nearby populations indicating minimal population divergence between these colonies.

**Figure 5:**
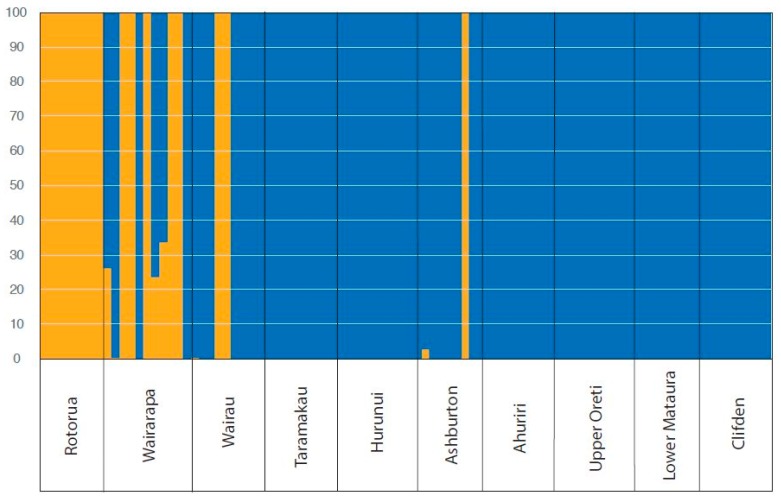
Visualisation of the optimal number of clusters determined by fastSTRUCTURE [49] to explain genetic structure seen in black-billed gull SNPs across New Zealand. Labels on the *x*-axis correspond to individual catchments sampled.

The optimal number of clusters as determined in fastSTRUCTURE that maximises marginal likelihood was k = 1; however, the optimal model components that best explained structure in the data was k = 2. When these results were visualised, the second cluster revealed that Wairau and Ashburton contributed to some of the ancestry of the North Island birds (Figure 5).

## 4. Discussion

The two major findings from this study are that (1) there is mito-nuclear divergence in some black-billed gulls, due to historical introgression with red-billed gulls, and (2) there is low but significant population structure within black-billed gull breeding colonies across the country. A pattern of IBD is seen across their range, showing increasing isolation and lower diversity for the breeding populations in the North Island (particularly in Rotorua). These observations help us understand the spatial and breeding ecology of black-billed gulls, with important implications for the species’ management.

### 4.1. Mito-Nuclear Discordance

In our study of black-billed gull genetic structure, results from mtDNA markers were discordant with nuclear DNA markers (SNPs). Mitochondrial DNA control region analyses showed no genetic structure between populations. The two major clades in the Bayesian tree and haplotype network (Figure 1 and Figure 2) were also seen in the analysis of a small sample of cytochrome b (Figure S1). Since the sequences in the smaller of the two major clades in both the control region and cytochrome b were nearly identical to the red-billed gull, mitochondrial introgression is likely to have occurred between these two species. Subsequent to this hybridisation event, these introgressed mitochondrial lineages have moved to various locations, removing a spatial signal in this data. The SNP analyses of the nuclear DNA showed differentiation between the North Island populations (particularly Rotorua) and the remaining populations, and a general pattern of IBD for the South Island colonies.

Many factors influence genetic structure, such as gene flow, IBD, historical fragmentation, range expansion, long-distance colonisation, land/ice barriers, or non-breeding distribution [2,59]. For example, natal dispersal, the movement of young birds between fledging and first breeding, and breeding dispersal, the movement of adults between breeding seasons, affect gene flow and population structuring [60]. Factors affecting dispersal are complicated but likely involve the behaviours of individuals and the conditions at each breeding site which affect prospecting young birds [2]. If the local environment is poor, failed breeders may move elsewhere and young prospecting birds may follow [2]. Black-billed gulls in Southland have high natal dispersal beyond their catchments, probably due to the highly unstable breeding habitat where natal colonies may not re-form over two consecutive seasons [16]. The likelihood of natal dispersal decreased with distance but there was infrequent long-distance dispersal from Southland to Otago and Canterbury regions. Breeding dispersal out of catchments was also found, although this was not as far or as common as natal dispersal. Additional findings by [16] included no evidence of group adherence as adults or chicks, the availability of a site used in the previous season did not result in the re-establishment of a colony at that site, and poor breeding success in one season did not cause abandonment of that site in the following season. The mtDNA results present here agree with the results of [16] because natal and breeding dispersal across catchments, lack of group adherence, and shifting colony locations would all result in gene flow.

Seabirds typically cover vast distances to find food sources thereby increasing the chance of population mixing and hence gene flow and genetic structure [61]. Contact between distant populations in non-breeding grounds may lead to individuals moving to a different breeding site; however, if populations have specific non-breeding sites or if they remain near breeding colonies, chances of an encounter with other populations are reduced [2,62]. For example, grey-headed albatrosses (*Thalassarche chrysostoma*) showed no genetic structure in mtDNA or nuclear DNA because they forage and disperse over large areas during the non-breeding season [62]. Black-browed albatrosses (*Thalassarche melanophris*) formed three genetic groups, because they use similar areas during the breeding and non-breeding season [63]. Black-billed gulls reside only in New Zealand, and populations have a high probability of mixing, particularly during the non-breeding season, due to the comparatively small land area of New Zealand. The gulls are known to move to the coast during the winter, and roost in large flocks [6]. This would support the lack of population structure seen when examining mtDNA markers.

Single nucleotide polymorphisms are found throughout the genome, and when genotyped in large numbers reveal high resolution estimates of population structure and connectivity, such as was found by reference [64] in white-chinned petrels (*Procellaria aequinoctialis*). The nuclear DNA analyses presented here show population structure with two clear clusters, Rotorua (Bay of Plenty region) as one group, and the remainder of the populations as another (Figure 3, Figure 4 and Figure 5). Wairarapa (Wellington region) shows intermediate differentiation, but not enough to be clearly distinct (Figure 4). Since Wairarapa lies between the South Island and Rotorua, this divergence supports the suspected northwards movement of black-billed gulls [14]. The South Island has lower pairwise F_ST_ values than among the North Island sites, which is indicative of genetic similarities between the South Island birds (Table 3). IBD is evident in the South Island which does not form clear genetically distinct clusters (Figure 4 and Figure 5). Overall, this pattern reveals that (1) there is lower connectivity between the North Island and the South Island, and also between the Rotorua and Wairarapa populations, than there is among the South Island populations, and (2) black-billed gulls are slightly philopatric throughout their range, with gene flow primarily occurring between nearby colonies. Black-billed gulls are therefore not panmictic across New Zealand, with gene flow primarily occurring in a stepwise system across the landscape.

Available South Island banding data for black-billed gulls confirms both small and large-scale movements. There are records of birds showing site fidelity, while others are known to move between colonies and catchments after breeding [17]. Young birds from the South Island’s West Coast region have also been seen on the east coast (Canterbury region) during the winter [65]. Birds from Marlborough have been confirmed to move to the Manawatu-Whanganui and Wairarapa regions of the North Island [17]. One individual banded at the Clarence River (northern Canterbury) moved to the Firth of Thames (Auckland/Waikato region, North Island) during the winter and returned to the Clarence in the summer [17]. These banding results, as well as those described above from reference [16], indicate mixing of colonies thereby supporting the minimal structure observed in the nuclear DNA of the South Island colonies, and inter-island movement. Available banding records from the North Island are sparse and show minimal movement between North Island colonies [66,67]. Genetic analyses would need to be done on Auckland and Taupo colonies to determine more detailed North Island population connectivity.

Due to the difference in effective population size for mtDNA versus nuclear DNA, mtDNA undergoes sorting and loses ancestral polymorphisms faster than nuclear DNA [20,68]. Since black-billed gulls did not breed at Rotorua until the 1930s, the structure in the slower evolving nuclear DNA, but not in the faster evolving mtDNA, is likely due to founder events. SNP analyses are very sensitive to population size decreases, and Rotorua is one of the most recently founded sampled populations. The genetic structure of the Rotorua colony could also be due to the hybridisation of red-billed and black-billed gulls seen in mtDNA analyses (introgression, discussed below), which could have also affected the nuclear DNA, most strongly in the Rotorua birds. The full influence of red-billed gull genetics in the nuclear genome of black-billed gulls remains unclear and beyond the scope of this study. However, given the higher pairwise relatedness and decreased diversity in this population, it appears that founder effects and low connectivity are primarily responsible for the observed patterns.

### 4.2. Introgression

Since the nuclear DNA showed geographic population structure, whereas the mtDNA did not, we used cytochrome b sequencing to rule out the possibility that geographic structure was overlooked because we were detecting the presence of nuclear copies of mitochondrial control region (NUMTs) [69]. Although rare, unintentional sequencing of a nuclear copy of a mtDNA region is possible [69]. Cytochrome b analyses (Figure S1) showed the same structure as the control region Bayesian tree (Figure 1) thereby reducing the possibility of NUMTs. Also, there were no stop codons or shifts in reading frame in the cytochrome b, which are indicative of NUMT gene regions [70,71]. Since sequences in the smaller of the two major clades in both the control region and cytochrome b aligned closely and were nearly identical to those of the red-billed gull, introgression is the most likely interpretation of the data.

Introgression is the exchange of genetic material between two species through hybridisation [22] and is often observed through mito-nuclear discordance [68], as in our study. Hybridisation occurs in at least 10% of the world’s bird species [72,73], and is particularly common in *Larus* gulls where 22 of 42 species are known to hybridise [23,24]. Gulls also do not appear to be affected by postzygotic barriers, such as reduced viability or reproductive capacity, perhaps due to low genetic differences between taxa [23]. The small effective population size of mtDNA makes it more susceptible to the fixation of foreign haplotypes than nuclear DNA; consequently, introgression can be seen in mtDNA without any admixture in nuclear DNA [74]. In a recent genomic study of mice in New Zealand, for example, mitochondrial capture between subspecies was observed to have independently occurred in multiple populations with no detectable nuclear introgression [21].

The divergence between the two clades—the primarily red-billed gull clade and the primarily black billed gull clade is ~6%. Using the mitochondrial divergence rate of 2% per million years as recommended in several recent studies [75,76,77], these two species therefore diverged around three million years ago. This level of divergence is therefore considerable; though obviously occasional hybridisation has occurred over that history as indicated by the mitochondrial introgression.

The mechanisms behind introgression are complex, and many hypotheses have not been quantitatively tested [78]. Hypotheses include drift in small populations, different levels of gene flow, selective sweeps, spatial expansion, negative selection against introgression into the nuclear genome, and sex-biases [78,79]. Random drift in small populations with unidirectional backcrossing was used to explain the mito-nuclear discordance between *Larus marinus* and *Larus smithsonianus* [79]. Selective sweeps occur when an advantageous haplotype replaces other haplotypes, and this can result in the loss of information on population structure and history [69]. Nuclear DNA can retain information because it has multiple markers, and the sweep is not likely to occur across several unlinked markers [69]. In a computer-simulated assessment of mito-nuclear introgression hypotheses by reference [78], sex-bias and spatial expansions were the least supported, and selective sweep with low dispersal rates the most supported. Determining the precise mechanisms behind the introgression observed among the black-billed and red-billed gulls is beyond the scope of this study; however, we are now aware of its existence.

Rapid introgression of an advantageous trait can aid the survival of a species [22] and can play an important role in evolution [74]. For example, a study on reproductive performance of hybrid and parental species of *Larus occidentalis* and *Larus glaucescens* showed that pure hybrid or mixed pairs had equal or better reproductive success than pure parental pairs, due to hybrid males having the best combined adaptive traits of both species [23]. For the black-billed gulls, it appears that for most of the individuals with discordant mitochondrial and nuclear DNA there has been minimal nuclear transfer from the red-billed gull, as they do not cluster away from the other individuals at nuclear loci. This phenomenon is known as mitochondrial capture, where mitochondria introgress between species, but nuclear DNA transfer is minimal or non-existent due to backcrossing [80]. This elimination of a nuclear signal of introgression can occur after only a few generations, and is surprisingly common [80,81].

### 4.3. Black Billed Gull Management and Future Research Recommendations

A decision on whether the North Island populations should be managed as a separate unit from the South Island needs to be considered. The Rotorua population appears to be the least connected and therefore most divergent population. It also appears to have the lowest diversity—indicating it probably was colonised from the south and now has comparatively low connectivity to the other breeding sites. This comparative isolation means that it will be the least likely to be rescued by immigrants from other populations should it decline. However, the North Island holds only 1.6% of the total population, and the importance of these birds from a genetic perspective needs to be evaluated further. There appears to have been recent expansion into North Island breeding sites. This is unlikely to be from human mediated gene flow, as people are unlikely to have moved them deliberately, and black-billed gulls are not as closely “connected” or adapted to human activities or things like landfills as other species of gulls are (i.e., black-backed gulls). It is however possible that anthropogenic forest clearance has created increased habitat in the North Island facilitating a northwards expansion. For the South Island, sufficient movement between colonies and regions appears to be taking place to allow for gene flow, although this is most likely to occur from nearby colonies. It is therefore not necessary to focus on the protection or intensive management of single specific colonies, but rather to manage these populations within a metapopulation context [82].

Future studies should focus on a more detailed analysis of the genetic structure of the North Island colonies (i.e., connections between Wairarapa, Rotorua, Taupo, and Auckland birds; the latter were not sampled here). This would involve sampling a larger number of North Island colonies (particularly Auckland) than what was done in our study and identifying movements and gene flow among colonies along the northern edge of the black-billed gulls’ range. The mechanisms of the hybridisation between red-billed and black-billed gulls should be more closely studied, both genetically and with direct observations. This future research could also then examine what nuclear genes have introgressed from red-billed gulls into black-billed gulls, and potentially reveal genes related to hybrid compatibility, or differential adaptation [23].

## Figures and Tables

**Table 1:** Genetic variation at 597 bp of mtDNA control region (first domain) in New Zealand black-billed gull populations, calculated using only the individuals from the larger of the two main clades, shown in Figure 1, to reduce any potential bias caused by hybridisation with red-billed gulls (individuals in the smaller of the two main clades).

Island	Region	Area/River	*n*	*h*	*ɧ*	*π*
North	Bay of Plenty	Rotorua	6	6	1.000	0.006
	Wellington	Wairarapa	7	4	0.810	0.006
Overall North	-	-	13	7	0.872	0.006
South	Marlborough	Wairau	7	4	0.714	0.007
	West Coast	Taramakau	5	3	0.700	0.003
	Canterbury	Hurunui	7	7	1.000	0.008
		Ashburton	6	5	0.933	0.005
		Ahuriri	4	4	1.000	0.011
	-	-	17	14	0.971	0.008
	Southland	Oreti	7	7	1.000	0.007
		Mataura	3	3	1.000	0.008
		Clifden	6	5	0.933	0.009
	-	-	16	13	0.975	0.008
Overall South	-	-	45	24	0.927	0.007
Total Overall	-	-	58	25	0.915	0.007

*n*: sample size, *h*: number of haplotypes, *ɧ*: haplotype diversity, *π*: nucleotide diversity.

**Table 2:** Genetic diversity indices for black-billed gulls at each sampling site. Ho-observed heterozygosity, Hs-expected heterozygosity, AR—allelic richness. Rot—Rotorua, Wairap—Wairarapa, Wai—Wairau, Tara—Taramakau, Huru—Hurunui, Ash—Ashburton, Ahu—Ahuriri, Ore—Oreti, Mat—Mataura, Cli—Clifden.

	Rot	Wairap	Wai	Tara	Huru	Ash	Ahu	Ore	Mat	Clif	Population Combined
Ho	0.258	0.287	0.227	0.286	0.278	0.258	0.244	0.252	0.280	0.263	0.2633
Hs	0.271	0.283	0.279	0.286	0.281	0.282	0.283	0.284	0.288	0.284	0.2818
AR	1.676	1.713	1.705	1.731	1.714	1.713	1.716	1.722	1.735	1.724	1.715

**Table 3:** Black-billed gull genetic differentiation matrix (F_ST_) between catchments across New Zealand from SNPs. Bold indicates significant differences from 0 (*p* < 0.05). Ahu—Ahuriri, Ash—Ashburton, Huru—Hurunui, Wai—Wairau, Tara—Taramakau, Ore—Oreti, Cli—Clifden, Mat—Mataura, Wairap—Wairarapa, Rot—Rotorua.

	Ahu	Ash	Huru	Wai	Tara	Ore	Cli	Mat	Wairap
Ahu									
Ash	0.006								
Huru	**0.004**	0.002							
Wai	0.005	0.005	0.002						
Tara	0.004	**0.007**	**0.004**	**0.006**					
Ore	0.005	**0.010**	**0.007**	**0.009**	**0.007**				
Cli	**0.006**	**0.010**	**0.009**	**0.011**	**0.007**	0.003			
Mat	0.004	**0.008**	**0.007**	**0.006**	**0.005**	0.002	0.003		
Wairap	**0.014**	**0.015**	**0.011**	**0.015**	**0.014**	**0.020**	**0.022**	**0.017**	
Rot	**0.033**	**0.035**	**0.028**	**0.035**	**0.033**	**0.037**	**0.043**	**0.037**	**0.021**

## Supplementary Materials

The following are available online at http://www.mdpi.com/2073-4425/9/11/544/s1; Text S1: Methods for mtDNA control region primer design and sequencing; Text S2: Methods for mtDNA cytochrome b sequencing; Text S3: Results for mtDNA cytochrome b; Figure S1: Neighbour-joining tree of mitochondrial cytochrome b using a small selectin of black-billed gulls sampled from across New Zealand. Figure S2. Isolation by distance (IBD) plots showing linearised F_ST_ vs Ln(distance) for (A) all sampling locations, and (B) only the South Island sites.
