# Supplementary material for: Population Connectivity and Traces of Mitochondrial Introgression in New Zealand Black-Billed Gulls (Larus bulleri)
Source: Genes (Basel). 2018 Nov 9;9(11):544. doi: 10.3390/genes9110544 (PMC6266082; doi:10.3390/genes9110544)
Supplement: Supplementary file 1 [file genes-09-00544-s001.pdf]

Supplementary material

Methods for mtDNA Control Region Primer Design and Sequencing

A phenol-chloroform extraction was used to extract genomic DNA from 100 blood samples [31] chosen from 10 colonies across the range (Figure 1). To aid primer design to access the control region, the total purified genomic DNA from one blood sample was sequenced on the Illumina platform (MiSeq 2X250 PE, V2) by New Zealand Genomics Ltd. The DNA reads from this single sample were BLAST searched using GENEIOUS software (v.9.0.5, Biomatters, New Zealand) against 2,043 bp mitochondrial region containing partial ND6 gene, full control region, and partial 12S gene of the black-headed gull (*Chroicocephalus ridibundus*; NCBI Accession No: FM209696.1). A total of 34 black-billed gull reads were identified, which included 22 reads covering 659 bp of the central black-billed gull control region. A primer pair to amplify the entire control region (*c.* 1200 bp) from the ND6 gene to the 12S gene was designed with Primer3 (web version, http://primer3.ut.ee) using the identified reads for the ND6 and 12S genes. The primers were BBG_CR_ND6_1F (5′-CCCCAGAACAAAACACAACC-3′) and BBG_CR_12S_1R (5′-CCCGCTCCTCTCTCCTTAGT-3′). The control region fragment was amplified in a 25 μL reaction volume containing 25ng of DNA, 1 x NH_4 buffer, 1.5 mM $MgCl_2$, 200 mM dNTPs, 0.4 pmol of each primer, and 0.5 units of *Taq* polymerase (Bioline USA Inc, Randolph, MA, USA) or in a 10 μL reaction volume containing 15ng of DNA, 10 μM of each primer, and 2 units of MyFi Mix (Bioline USA Inc, Randolph, MA, USA). Thermal cycling consisted of 5-min denaturation at 94°C, 10 touchdown cycles of 94°C/30 sec, 62°C→52°C/30 sec (decrease of 1°C per cycle), 72°C/90 sec, finishing with 30 cycles of 94°C/30 sec, 50°C/30 sec, and 72°C/90 sec. Purified PCR products (Acroprep 96 filter plates, PALL Corporation, Port Washington, NY, USA) were sequenced (BigDye Terminator v.3.1, ThermoFisher, Waltham, MA, USA) on an ABI 3730xl DNA analyser (Applied Biosystems Inc., Foster City, CA, USA). Due to difficulty sequencing through the control region "goose hair pin" and a poly-T stretch (*c.* 10 bp) in the center of the control region with the ND6 and 12S primers, two internal control region primers, BBG_CR_INT_R1 (5′-GCCCTGACATAGGAACCAGA-3′) and BBG_GOOSEHAIRPIN_F1 (5′-ACATCCCTCCCCAACACAT-3′) were used to sequence a 597 bp fragment of the first domain of the control region.

Sequences were aligned with Geneious® 9.0.5. Variable sites in consensus sequences were verified by visual inspection of chromatograms. Of the 100 extracted DNA samples, 69 samples were able to be amplified with sequences produced. The 1,207 bp D-loop region sequence of the common gull (*Larus canus*) was used as an outgroup (NCBI Accession No: AB208768.1) and trimmed to fit the sequence lengths of the black-billed gulls following alignments done in Geneious®. The 221 bp D-loop domain region I sequence of the red-billed gull (NCBI Accession No: AY584133.1) was trimmed to fit the sequence lengths of the black-billed gulls following alignments done in Geneious®, and solely used as a basis for determining how closely it matched the black-billed gull. Equally, a published 430 bp *L. bulleri* control region sequence (NCBI Accession No: FM209657) was adjusted and aligned for comparisons.

Methods for mtDNA cytochrome b sequencing

Samples were chosen from Ashburton, Ahuriri, Clifden, and Taramakau to match the control region samples of the small clade. A ~1,000 bp fragment containing the entire cytochrome b gene was amplified using PCR primers H16064 (5′-CTTCAGTTTTTGGTTTACAAGACC-3′) and L14764 (5′-TGRTACAAAAAAATAGGMCCMGAAGG-3′) [33]. A 10 μL reaction volume containing 15ng of DNA, 10 μM of each primer, and 2 units of MyFi Mix (Bioline USA Inc, Randolph, MA, USA) was used for amplification, and thermal cycling consisted of 5-min denaturation at 95°C, 10 touchdown cycles of 95°C/20 sec, 64°C→54°C/25 sec (decrease of 1°C per cycle), 72°C/70 sec, finishing with 25 cycles of 95°C/20 sec, 50°C/25 sec, and 72°C/70 sec. Purified PCR products (Acroprep 96 filter plates, PALL Corporation Port

Washington, NY, USA) were sequenced (BigDye Terminator v.3.1, ThermoFisher, Waltham, MA, USA) on an ABI 3730xl DNA analyser (Applied Biosystems Inc., Foster City, CA, USA) with one primer, L14764.

Sequences were aligned with Geneious®. All seven samples were amplified and sequences produced, and aligned with a 1,143 bp cytochrome b sequence of the red-billed gull (NCBI Accession No: FM209918) and a 1,143 bp cytochrome b sequence of a black-billed gull (NCBI Accession No: FM209900.1) as comparisons and to determine the presence of introgression. This alignment was used to draw a neighbour-joining tree in Geneious®.

Results for mtDNA cytochrome b

The four birds chosen from the small clade in Figure 1 (BBG002_Ash, BBG014_Ahu, BBG043_Tara, BBG023_Clif) were aligned with the published 1,143 bp cytochrome b sequence of the red-billed gull (NCBI Accession No: FM209918; sequence lengths trimmed to 868 bp) with a close match of only one bp difference between the 4 sequenced individuals and the published sequence (Figure S1). The three birds chosen from the large clade in Figure 1 (BBG009_Ash, BBG012_Ahu, BBG029_Clif) were aligned with the published 1,143 bp cytochrome b sequence of a black-billed gull (NCBI Accession No: FM209900.1; sequence lengths adjusted to 869 bp) with a close match of only one bp difference between the three sequenced individuals and the published sequence (Figure S1).

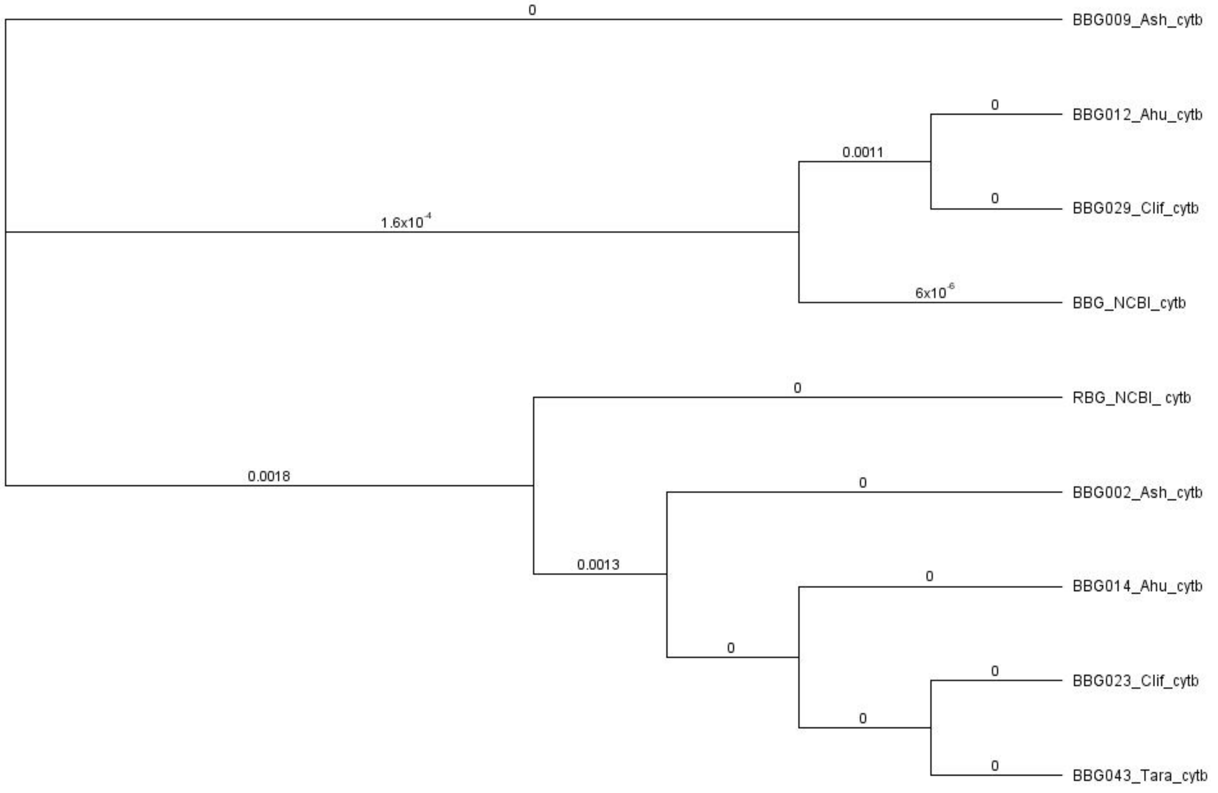

Figure S1. Neighbour-joining tree of mitochondrial cytochrome b using a small selection of black-billed gulls sampled from across New Zealand. Posterior probabilities are shown above the branches.

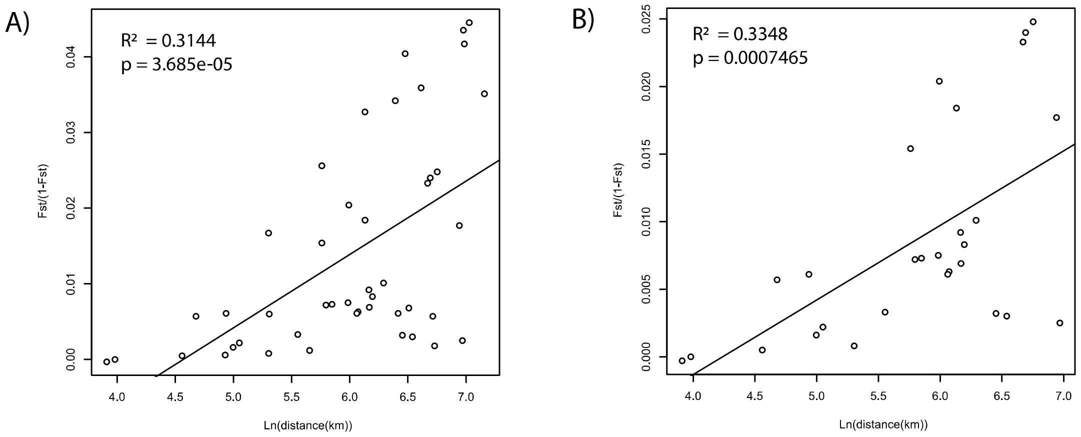

Spearman's rank correlation
S = 6057.5, p-value = 1.271e-05
rho = 0.6009554

Spearman's rank correlation
S = 1407.7, p-value = 0.0004998
rho = 0.614753

Figure S2. Isolation by distance (isolation-by-distance—IBD) plots showing linearized F_{ST} vs Ln(distance) for (**A**) all sampling locations, and (**B**) only the South Island sites.
